# Prevalence of Atrial Fibrillation in Semiurban Nepal: Result From a Community-Based Cross-Sectional Screening

**DOI:** 10.1155/2024/1759135

**Published:** 2024-10-28

**Authors:** Rojeena Koju Shrestha, Durga Bista, Rajani Shakya, Rajendra Prasad Koju, Ram Bahadur Gurung

**Affiliations:** ^1^Department of Pharmacy, Kathmandu University, Dhulikhel, Kavre, Nepal; ^2^Department of Internal Medicine, Dhulikhel Hospital, Kathmandu University School of Medical Sciences, Dhulikhel, Kavre, Nepal

**Keywords:** atrial fibrillation, community screening, oral anticoagulant

## Abstract

**Background:** Atrial fibrillation (AF) is a common morbid arrhythmia that can cause thromboembolic events such as stroke. Despite advancements in diagnostic technologies, a significant number of AF patients may remain undetected and undiagnosed, and these asymptomatic patients possess sufficient risk of cardioembolic stroke. Identifying such patients through appropriate screening techniques and timely initiation of systemic anticoagulation therapy is essential to prevent such life-threatening complications.

**Objectives:** The objectives of this study encompass screening of AF among residents of the Dhulikhel Municipality and identifying its prevalence, along with evaluation of stroke risk and use of antithrombotic therapy in patients confirmed with AF.

**Methods:** All residents of four wards of Dhulikhel Municipality, aged 50 years and above (*n* = 2048), underwent one-time electrocardiogram (ECG) screening using a portable 12-lead ECG machine. The cardiologist checked the cardiogram, and suspected AF cases were referred to the hospital for further evaluation and appropriate management. They were followed up to find out information on disease confirmation and management.

**Results:** Out of 2048 participants, AF was detected in 16 participants, resulting in an overall prevalence of 0.78% (CI 0.4%–1.3%). The prevalence of AF was highest (2.98%) in population aged 80 years and above. Among individuals with AF, the median age was 71.5 (66.3–79.5) years, 50.0% were male and 75.0% had high stroke risk as indicated by a CHA_2_DS_2_-VASc score ≥ 2. Among these patients, only 41.66% were treated with oral anticoagulants (OACs), while 58.34% were treated either with single or dual antiplatelet therapy (DAPT).

**Conclusion:** This study provided important insight into the prevalence of AF at the community level. Many AF patients were at high risk of stroke, but the OAC use was less than 50%. Screening of AF needs to be carried out on a larger scale in Nepal for early detection and timely management of the disease.

## 1. Introduction

Atrial fibrillation (AF) is the most common sustained cardiac arrhythmia, increasing its prevalence with age [1]. Globally, the prevalence of AF is approximately around 0.51% [2]. AF prevalence has been observed to be higher in developed countries such as Australia, Europe, and the United States of America (1%–4%) [3–9], while it is comparatively low in Asian countries including India and China (0.3%–2.8%). In Nepal, it ranged from 0.66% to 13.80% [10–12]. However, a considerable number of people with AF remained undiagnosed and untreated due to asymptomatic or paroxysmal AF [13].

AF is associated with an increased risk of stroke, heart failure, and cardiovascular mortality [14]. Patients with AF had a fivefold greater risk of ischemic stroke, a threefold increased risk of heart failure, and 3.5 fold increased risk of mortality [15, 16]. Many contemporary guidelines recommend the use of the CHA_2_DS_2_-VASc score (congestive heart failure, hypertension, age ≥ 75 years [doubled], diabetes, prior stroke or TIA [doubled], vascular disease, age 65–74 years, and sex category [female]) as a common stroke risk stratification tool for the predicting stroke in patients with AF [17, 18]. A CHA_2_DS_2_-VASc score ≥ 2 in males and ≥ 3 in females is considered a high risk of stroke [19]. A majority of AF patients ranging from 59.0% to 70.9% had a high risk of stroke [5, 20, 21]. AF patients with a CHA_2_DS_2_-VASc score of two or more are recommended for oral anticoagulation (OAC) therapy to reduce the risk of stroke [15, 17, 22]. Some studies reported that approximately half of AF patients eligible for OAC therapy were treated as per standard treatment guidelines recommendation [21, 23, 24]. Another study identified that one-third of the AF cases were found to be undertreated [24]. The use of OAC (58.92%) in high-risk patients is increasing in our country but is still underused [21] Furthermore, more than 50.0% of AF patients are still kept under antiplatelet therapy [25] which is now no longer suggested for stroke prevention [15].

Considering the burden of morbidity and mortality as a consequence of AF, community screening for this condition can be of great value. Cases of stroke can be avoided by implementing screening programs in the general population for early identification and initiation of OAC therapy which helps to reduce the burden of mortality and morbidity [2, 13, 26]. The screening program at a single time point can be used to detect previously undiagnosed AF, regardless of the screening site [20].

The European Society of Cardiology (ESC) recommends AF screening by pulse palpation or 12-lead electrocardiogram (ECG) [15]. But there are other tools such as modified blood pressure monitors, single-lead ECG, and 3-lead ECG for detecting AF. Newer technologies such as smartphones, watch devices, and handheld devices are also available. Even though these tools offer initial detection, 12-lead ECG interpreted by a cardiologist is considered a gold standard for the confirmation of the AF [27] and was used for screening in the present study.

Limited data are available on the prevalence of AF, the proportion of AF patients at high risk of thromboembolic events, and utilization of OAC therapy in Nepal. To fill the gap, this study was designed to screen community people, assess the stroke risk, and evaluate the use of antithrombotic therapy in the diagnosed patients.

## 2. Methods

This community-based cross-sectional study was carried out among the permanent residents of Dhulikhel Municipality, aged 50 years and above. Municipality is an administrative division in the provinces of Nepal which is further divided into wards, the smallest unit of local government. Dhulikhel Municipality situated in Kavre District, Nepal, comprises 13 wards. Out of these 13 wards, 4 different wards were randomly selected for data collection. The residents of these wards comprised the base population for the study. A voter list, which is a registered list of all the adults over the age of 18 years eligible to vote in specific locality, was obtained from the municipality office. Residents consenting to participate and fulfilling the set inclusion criteria were enrolled in the study.

The research assistants, nurses, and pharmacists, who were familiar with the layout of the survey sites, were recruited. They were trained by a cardiologist and nursing staff of the Dhulikhel Hospital, which is a university teaching hospital. The training provided an overview of the disease and its management with a special focus on the operation of the ECG and the interpretation of collected data.

### 2.1. Data Collection

Among the randomly selected wards, the residents of age ≥ 50 years and who consented to participate were included in the study after obtaining written informed consent. One-time AF screening was conducted by the trained research assistant from a period of March to December 2022. The screening activities took place either by visiting the participants' homes or by inviting them to the primary healthcare centers in their respective wards. Community social workers were also involved in disseminating information about the screening program. The residents were excluded if they were suffering from terminal illnesses, not staying in the stated ward, or were not willing to participate in the study.

The data collectors obtained participants' sociodemographic information and history of comorbidities from past medical records, clinical data including height, weight, body mass index (BMI), blood pressure, and 12-lead ECG with automated analysis. The ECG was recorded for 25 mm/sec, and the record was subsequently interpreted by a cardiologist for possible AF. Suspected cases were referred to Dhulikhel Hospital for the final diagnosis and management upon confirmation. They were followed up to find out about disease confirmation and management.

The stroke risk of the confirmed patients was assessed by using the CHA_2_DS_2_-VASc score. The CHA_2_DS_2_-VASc score of zero in males and one in females were categorized as “low-risk,” with a score of one in males and two in females as “moderate risk,” and a score of two in males and three in females as “high risk”. The use of antithrombotics for the treatment of AF was studied, and an appropriate use of OAC therapy was assessed based on stroke risk stratification. AF patients with CHA_2_DS_2_-VASc score ≥ 2 are considered appropriate for the use of anticoagulation therapy in this study.

### 2.2. Study Outcomes

The outcomes of the study were the prevalence of AF, risk of stroke, and utilization of antithrombotic therapy of the confirmed AF patient detected at community screening.

### 2.3. Ethical Considerations

The ethical approval was obtained from the Nepal Health Research Council (ref no 806) and Institutional Research Committee (IRC), Kathmandu University, School of Medical Sciences, Dhulikhel (ref no 104/2021). Furthermore, approval was obtained from Dhulikhel Municipality.

### 2.4. Data Analysis

The collected information was initially documented on paper-based forms. Later, the data were transferred to an electronic database for further analysis. The data management process ensured the confidentiality and privacy of participant information.

Data were then analyzed using SPSS version 23.0. All continuous variables were tested for normality by using the Wilk test. Variables with normal distribution were expressed as mean and standard deviation and tested for differences with Student's *t*-test. Non-normal data were presented as medians and interquartile ranges (IQR), and differences were tested with the Mann-Whitney U test. *p* value < 0.05 was considered as statistically significant. Categorical variables were presented as counts and percentages, and groups were compared using *χ*^2^ tests or Fisher's exact tests for small samples.

## 3. Results

**Figure 1:**
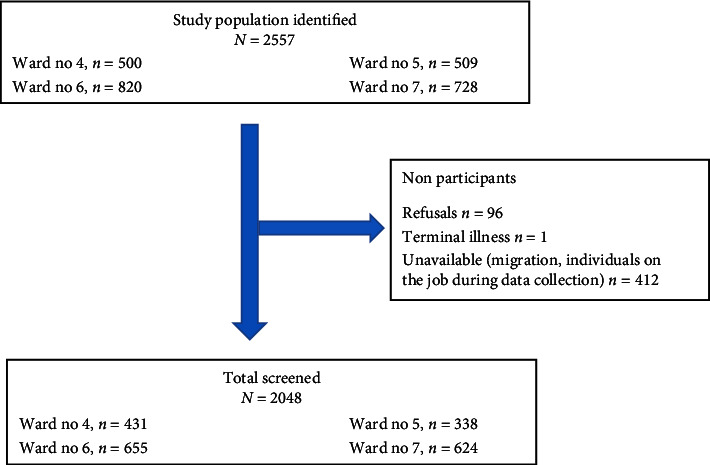
Schematic diagram of study population enrollment.

A total of 2557 residents aged 50 years and above were identified from the voter list of four wards of Dhulikhel Municipality. Over nine months, 2048 eligible residents out of 2557 were screened for AF. The remaining individuals were not included in the study either due to their lack of interest in participating in the screening or unavailable due to their migration to other places. The enrollment details of the study population for AF screening are presented in Figure 1.

An overview of the total study population and their categorization into two groups: “without AF” (*n* = 2032) and “with AF” (*n* = 16), are summarized in Table 1.

The median age of the participants was 61 years with the majority (43.5%) belonging to the age group 50–59 years. More than half of the participants were female (56.9%), and most of them had not received formal education (53.3%). Two-fifths of the participants were overweight (40.6%), and most of them were nonsmokers and nonalcoholics, 81.8% and 85.4%, respectively. The median systolic blood pressure (SBP), diastolic blood pressure (DBP), and heart rate (HR) were 130 mm Hg, 80 mm Hg, and 77 beats per minute, respectively. A significant number were suffering from various disease conditions (57.3%) with the most prevalent being hypertension (40.6%) followed by diabetes mellitus (17.9%). The median age and prevalence of hypertension in participants with and without AF were found to be significantly different. Besides this, there were no significant differences in other characteristics as shown in Table 1.

**Figure 2:**
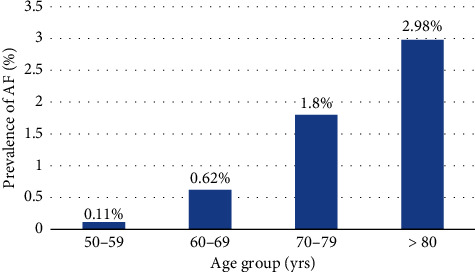
Age-stratified prevalence of AF.

The overall prevalence of AF was 16 (0.78%, CI 0.4%–1.3%) among the screened participants. Among these 16 patients, eight were newly diagnosed with AF while eight had a previous history of AF, resulting in a prevalence of 0.39% (CI 0.2%–0.8%) for each group. The prevalence of AF was highest (2.98%, CI 0.08%–7.5%) in the population aged 80 years and above (Figure 2).

**Figure 3:**
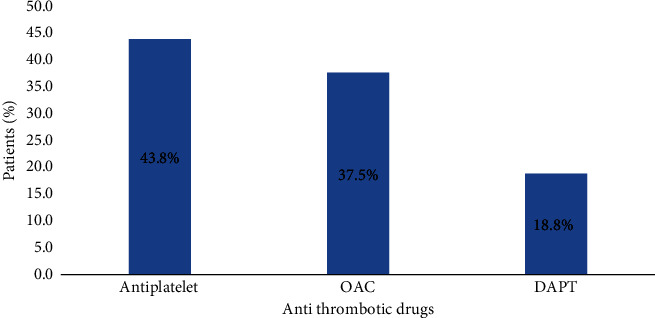
Antithrombotic treatment in AF patients.

Aspirin was prescribed in 43.8% of patients, whereas OAC was prescribed in 37.5% of patients (Figure 3).

**Figure 4:**
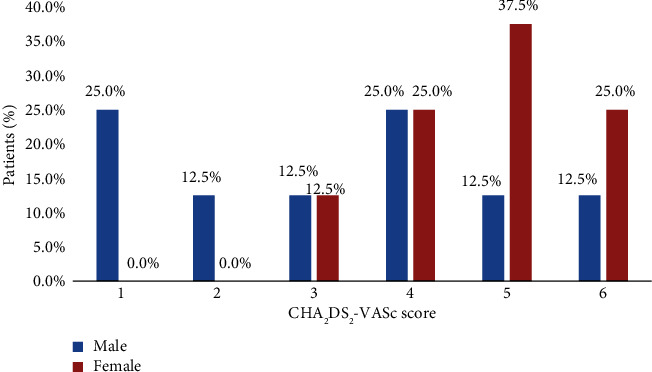
Distribution of CHA_2_DS_2_-VASc score in male and female patients (*n* = 16).

Among male patients with AF, the majority (25.0%) had CHA_2_DS_2_-VASc scores 0 and 3, whereas the majority (87.5%) of females with AF had scores 3 and above (Figure 4).

**Figure 5:**
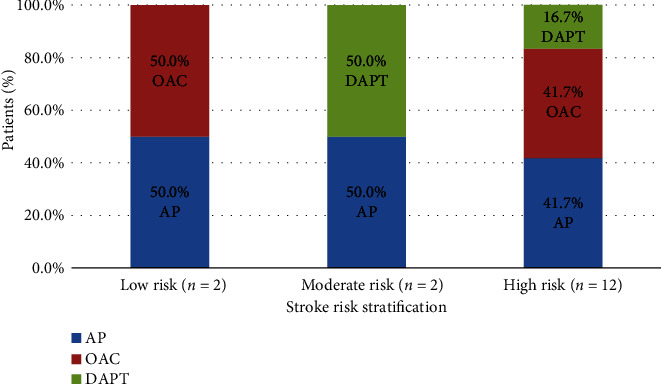
Antithrombotic therapy according to the stroke risk stratification. AP, antiplatelet; OAC, oral anticoagulants; DAPT, dual antiplatelet. Low risk: CHA_2_DS_2_-VASc score of zero in males and one in females; moderate risk: a score of one in males and two in females; high risk: a score of two in males and three in females.

The majority of the patients (75.0%) had CHA_2_DS_2_-VASc score ≥ 2, indicating a high risk of stroke. Among those patients, only 41.7% were on the OAC therapy, and the remaining (58.3%) were on either single or dual antiplatelet therapy (DAPT) reporting guideline nonadherence in antithrombotic therapy (Figure 5).

## 4. Discussion

The present study found the prevalence of AF in the community of Dhulikhel, Kavre District, Nepal, to be 0.78%. This finding aligned closely with the findings from other studies conducted in different settings. In a study conducted in the internal medicine department of the tertiary care hospital of Biratnagar, Nepal, the prevalence was reported to be 0.66% [12]. Likewise, our finding falls within the range of 0.49%–5.4% AF prevalence reported by studies conducted in different settings of various other Asian countries [4, 28–35]. However, our study had a higher prevalence compared to a study conducted in an Indian village, which reported a prevalence of only 0.1% in inhabitants ≥ 18 years [36]. These variations in prevalence rates among different studies can be attributed to differences in the study settings and the age groups of the people included in the studies. Canty et al. reported that the prevalence of newly diagnosed AF ranges from 0% to 34.5% [37]. The prevalence of newly diagnosed AF was 0.39% (CI 0.2%–0.8%) in the present study which falls within this range.

It was observed that the prevalence of AF increased with age which was similar to other studies [5, 10] and was high (2.98%, 0.08%–7.5%) in participants aged > 80 years. Our study indicated an equal proportion of AF in male and female populations, which differs from other studies where the prevalence of AF was higher in males than females [32, 38]. This difference could be due to few number of diagnosed AF cases and the high number of female participants for screening in this study. Obesity (22.5%), alcohol consumption (14.6%), and smoking (18.6%) are known risk factors for AF that were detected in only a smaller proportion of AF patients which was similar to the study conducted in China [39]. Participants enrolled in this study were greater than 50 years and thus susceptible to various age-related chronic health conditions. This realization might have made them health conscious and avoid common risk factors such as alcohol and smoking, prioritizing a healthy lifestyle.

Furthermore, a substantial proportion of AF patients within our study cohort exhibited a predominant comorbidity of hypertension (40.6%), followed by diabetes mellitus (17.9%). These statistics were consistent with the findings of the study conducted in China [39] while slightly lower than the study of the Kerala registry (53.8%, 34.5%) [40]. In the J-RHYTHM registry of Japanese patients with valvular and nonvalvular AF, more than 50.0% of AF patients (59.1%) were found to have hypertension [41]. This contrast might be due to small and general population selected for the screening program in this study. These comorbid conditions have proven to be the modifiable risk factors that can predispose to AF [42]. Individuals with hypertension had a 1.7-fold greater risk of onset of AF and approximately one in six cases of AF can be attributed to hypertension [43]. Hypertension can elevate the risk of developing AF with left ventricular hypertrophy, diastolic dysfunction, and arterial atherosclerosis. These processes can ultimately contribute to the onset of atrial fibrosis and the emergence of arrhythmia [44]. Besides hypertension, studies also suggest diabetes mellitus as an independent risk factor for the development of AF. The presence of DM may also enhance progression from paroxysmal to persistent AF. The underlying mechanisms may be metabolic alterations inherent to diabetes mellitus including defects in hemostasis and fibrinolysis, increased angiogenesis, insulin resistance, and glucose intolerance. These changes result in the activation of the renin-angiotensin-aldosterone system (RAAS). The latter exerts an atherogenic and profibrotic stimulus on the cardiac muscle, forming the substrate for AF development [45].

A majority of patients were on antiplatelet therapy (43.8%) while a significant number (37.5%) of patients were on OAC. This finding was somewhat lower than other studies conducted by Sherpa et al. in Nepal (51.91% and 41.8%, respectively) [21]. This difference might be due to more elderly study population in our study, and increasing age is an independent risk factor for bleeding [46]. The data showed that a considerable number (75.0%) of the screened AF patients were at high risk of stroke, which was similar to the study carried out in another study carried out in Nepal (70.9%) [21] and India (79.0%) [5], indicating the necessity of OAC use as recommended in the current standard treatment guidelines [17, 47]. Despite this, our study findings revealed that only 41.7% of the patients at high risk of stroke were receiving OAC therapy, which was different from the value reported by the GARFIELD registry (59.3%) [48] and Sherpa et al. (58.9%) [21]. In a study conducted in a tertiary care hospital in Nepal, only one-fourth of the patients at high thromboembolic risk were being prescribed OACs, highlighting the underutilization of OACs in these populations [25]. Similarly, research conducted in the Korean populations identified a noteworthy proportion of AF patients who continued to be undertreated [49]. In contrast, it was observed in the EORP-AF pilot survey that among patients with CHA_2_DS_2_-VASc score ≥ 2, 70.9% received OAC [50]. Another study carried out in South Korea reported that nearly two-thirds of eligible patients who were recommended for OAC therapy were taking it [51]. Similar findings have been reported from the GLORIA-AF registry Phase II, highlighting a significant portion of individuals with AF treated with OAC [23]. Unfortunately, our study result showed a lower adherence rate to contemporary guidelines for OAC use for stroke prevention in AF compared to the result of the COOL-AF registry in 2020 reporting 68.1% guideline adherence [52]. Many high-stroke-risk patients are commonly treated with either single or dual antiplatelet medications. Despite antiplatelet therapy being not further recommended in stroke prevention by different current treatment guidelines [15, 53, 54], these medications continued to be prescribed. This apparent use of antiplatelet therapy and reluctance to prescribe OACs could be attributed to various factors. A majority of our patient populations were elderly, had a concern about the risk of falls which can lead to bleeding, and with multiple comorbidities. A vitamin antagonist (VKA) use is associated with the potential risk of bleeding and drug-drug interactions. There is a necessity for the determination of the international normalized ratio (INR) for VKAs and annual renal function tests for non-VKA OAC (NOAC) use [17]. In our specific context, many patients may face financial constraints that hinder their ability to afford regular diagnostic tests and purchase prescribed medications, particularly NOACs. Furthermore, logistic challenges, such as the inability to attend regular hospital follow-up might prohibit patients from receiving appropriate treatment as most of them are from remote areas. These all may contribute to the nonadherence of physicians to treatment guidelines. Some studies have highlighted the role of pharmacists in improving the use of OACs. Interdisciplinary work with pharmacists is important for improving the guideline-directed use of anticoagulants in stroke prevention in AF patients [55]. In addition, clinical pharmacist-led intervention reduced adverse effects related to the use of VKA [56]. Pharmacists' involvement in the direct OACs (DOACs) dose selection has also reduced the errors. Furthermore, medication reconciliation is done by pharmacists during hospital stays, at the time of discharge, and clinics may be able to decrease DOAC-related medication errors [57, 58]. Healthcare professionals and patients should discuss to identify and resolve any factors that may result in the undertreatment of disease so that the patients will receive appropriate and effective treatment.

## 5. Strength and Limitations

This screening program was provided free of charge to the participants. Therefore, there are no economic barriers that might have hindered participation in the study. Secondly, utilization of the 12-lead ECG machine is considered as the gold standard for the detection of AF which is the major strength of our study.

The limitations included the use of a single time point for screening that might have missed individuals with paroxysmal AF who were not in AF during screening. This study focused on certain wards of the Dhulikhel Municipality. Similarly, the study included a small population from the community of Dhulikhel. Therefore, the findings might not apply to the entire Nepalese population. The current study included participants aged 50 years and above, and the result cannot be generalized to the population aged below 50 years.

## 6. Conclusion

Community-based screening for AF utilizing a portable 12-lead ECG machine was feasible. The overall prevalence of AF observed in this study during community screening was less than 1%. Although the CHA_2_DS_2_-VASc score identified a significant number of AF patients at a high risk of thromboembolic events, it is noteworthy that a substantial portion of these individuals did not receive OAC therapy. Community-based screening can be conducted on a larger scale to identify individuals with AF so that early initiation of appropriate treatment can be done which will help to prevent the thromboembolic risk of AF. Furthermore, it is necessary to implement current treatment guidelines to enhance the use of OACs to prevent thromboembolic events in patients with AF.

## Figures and Tables

**Table 1:** Sociodemographic profile and clinical characteristics of the study population.

Characteristics	Total (*n* = 2048)	Without AF (*n* = 2032)	With AF (*n* = 16)	*p* value
Age (years) (median)	61.0 (54.0–70.0)	61.0 (54.0–70.0)	71.5 (66.3–79.5)	0.01^2^
Age category (years), *n* (%)				
50–59	890 (43.5)	889 (43.8)	1 (6.2)	0.001^1^
60–69	637 (31.1)	633 (31.2)	4 (25.0)	
70–79	387 (18.9)	380 (18.7)	7 (43.8)	
> 80	134 (6.5)	130 (6.4)	4 (25.0)	
Gender, *n* (%)				
Male	883 (43.1)	875 (43.1)	8 (50.0)	0.577
Female	1165 (56.9)	1157 (56.9)	8 (50.0)	
Education, *n* (%)				
No formal education	1092 (53.3)	1084 (53.3)	8 (50.0)	0.540
Less than high school	834 (40.7)	828 (40.7)	6 (37.5)	
More than high school	122 (6.0)	120 (5.9)	2 (12.5)	
BMI category, *n* (%)				
Underweight	69 (3.4)	68 (3.3)	1 (6.2)	0.677^1^
Normal	687 (33.5)	681 (33.5)	6 (37.5)	
Overweight	831 (40.6)	825 (40.6)	6 (37.5)	
Obese	461 (22.5)	458 (22.5)	3 (18.8)	
Smoking, *n* (%)				
Yes	372 (18.2)	371.(18.3)	1 (6.2)	0.332^1^
No	1676 (81.8)	1661 (81.7)	15 (93.8)	
Alcohol intake, *n* (%)				
Yes	300 (14.6)	296 (14.6)	4 (25.0)	0.276^1^
No	1748 (85.4)	1736 (85.4)	12 (75.0)	
SBP, median	130 (120–140)	130 (120–140)	130 (120–150)	0.572^2^
DBP, median	80 (70.0–90.0)	80 (70.0–90.0)	90 (72.5–97.5)	0.161^2^
HR, median	77 (69.3–85.0)	77 (69.3–85.0)	77 (76.8–90.0)	0.805^2^
Comorbid conditions, *n* (%)				
Hypertension	832 (40.6)	819 (40.3)	13 (81.3)	0.01
CAD	28 (1.4)	26 (1.3)	2 (12.5)	0.19^1^
Heart failure	3 (0.1)	3 (0.1)	0	1.000^1^
COPD	108 (5.3)	105 (5.2)	3 (18.8)	0.48^1^
Diabetes mellitus	366 (17.9)	359 (17.7)	7 (43.8)	0.14^1^
Dyslipidemia	240 (11.7)	236 (11.6)	4 (25.0)	0.108^1^
Stroke	3 (0.1)	2 (0.1)	1 (6.3)	0.23^1^
Hyperthyroidism	4 (0.2)	4 (0.2)	0	1.000^1^
Hypothyroidism	101 (4.9)	101 (5.0)	0	1.000^1^
TIA	2 (0.1)	2 (0.1)	0	1.000^1^

Abbreviations: BMI, body mass index; CAD, coronary artery disease; COPD, chronic obstructive pulmonary disease; DBP, diastolic blood pressure; HR, heart rate; SBP, systolic blood pressure; TIA, transient ischemic attack.

^1^Fisher's exact test.

^2^Mann-Whitney U test.

## Data Availability

Readers can get access to the data supporting the conclusions of the study by contacting the corresponding author.
